# Pathogen evolution during vaccination campaigns

**DOI:** 10.1371/journal.pbio.3001804

**Published:** 2022-09-23

**Authors:** Troy Day, David A. Kennedy, Andrew F. Read, Sylvain Gandon

**Affiliations:** 1 Department of Mathematics and Statistics, Department of Biology, Queen’s University, Kingston, Ontario, Canada; 2 Department of Biology, Pennsylvania State University, University Park, Pennsylvania, United States of America; 3 Department of Entomology, Pennsylvania State University, University Park, Pennsylvania, United States of America; 4 CEFE, CNRS, Univ Montpellier, EPHE, IRD, Montpellier, France

## Abstract

Following the initiation of the unprecedented global vaccination campaign against Severe Acute Respiratory Syndrome Coronavirus 2 (SARS-CoV-2), attention has now turned to the potential impact of this large-scale intervention on the evolution of the virus. In this Essay, we summarize what is currently known about pathogen evolution in the context of immune priming (including vaccination) from research on other pathogen species, with an eye towards the future evolution of SARS-CoV-2.

## Abstract 2

How might vaccination campaigns affect the evolution of viruses? This Essay summarizes what is currently known about viral evolution in the context of vaccination, with an eye towards the future evolution of SARS-CoV-2.

Adaptation of pathogens can occur when a novel variant is more fit in the current environment than its predecessors. Host immunity, whether generated by vaccination or natural infection, is one variable that shapes the current environment for pathogens. The scale of the current global vaccination campaign against Severe Acute Respiratory Syndrome Coronavirus 2 (SARS-CoV-2) and the speed at which new variants are arising has raised the question of how vaccination efforts might affect viral evolution.

It is useful to think of the temporal dynamics of evolutionary change for novel pathogens like SARS-CoV-2 as passing through 2 phases. In the first phase, the host population is immunologically naïve and selection strongly favors adaptation to these abundant naïve hosts. In the second phase, a growing proportion of the host population will have an immunological history with the pathogen, either through natural infection or vaccination, and thus selection will shift, increasingly favoring adaptation to these immune-primed hosts. In this Essay, we explore how pathogens in novel host species evolve in response to immunologically primed hosts, with a particular focus on vaccination.

## Conceptualizing pathogen evolution

Pathogen adaptation to naïve and primed hosts depends on the appearance of new variants as well as on their fitness in each host type. We can quantify fitness by considering both the absolute per capita growth rate of infections caused by a variant, as well as this growth rate relative to the growth rate of the currently dominant type (sometimes called the wild type). The absolute growth rate will determine if the variant can spread in a population, whereas the relative growth rate will determine if the variant can increase in frequency and thereby potentially displace the currently dominant type.

For a variant to spread in a population, its absolute growth rate must be positive (equivalently, its reproduction number must be larger than one). The absolute growth rate (*r*_*i*_) of infections caused by any pathogen variant (*i*) can be approximated as follows:

$$r_{i}=(1-p)r_{i,N}+pr_{i,P},$$ (1)

where (*p*) is the fraction of the population that has been primed against the pathogen, and (*r*_*i*,*N*_) and (*r*_*i*,*P*_) are the growth rates of infections by variant *i* in a fully naïve and fully primed population, respectively [1,2] (S1 Appendix).

For a variant to increase in relative frequency, and thus potentially displace the wild type, its selection coefficient (*s*), defined as the difference between its growth rate and that of the wild type, must be positive. For the above model, this selection coefficient is given by

$$s=(1-p){\Delta r}_{N}+p{\Delta r}_{P},$$ (2)

where (Δ*r*_*N*_) and (Δ*r*_*P*_) are the differences in growth rate between the variant and the wild type in a fully naïve and fully primed population, respectively.

**Fig 1:**
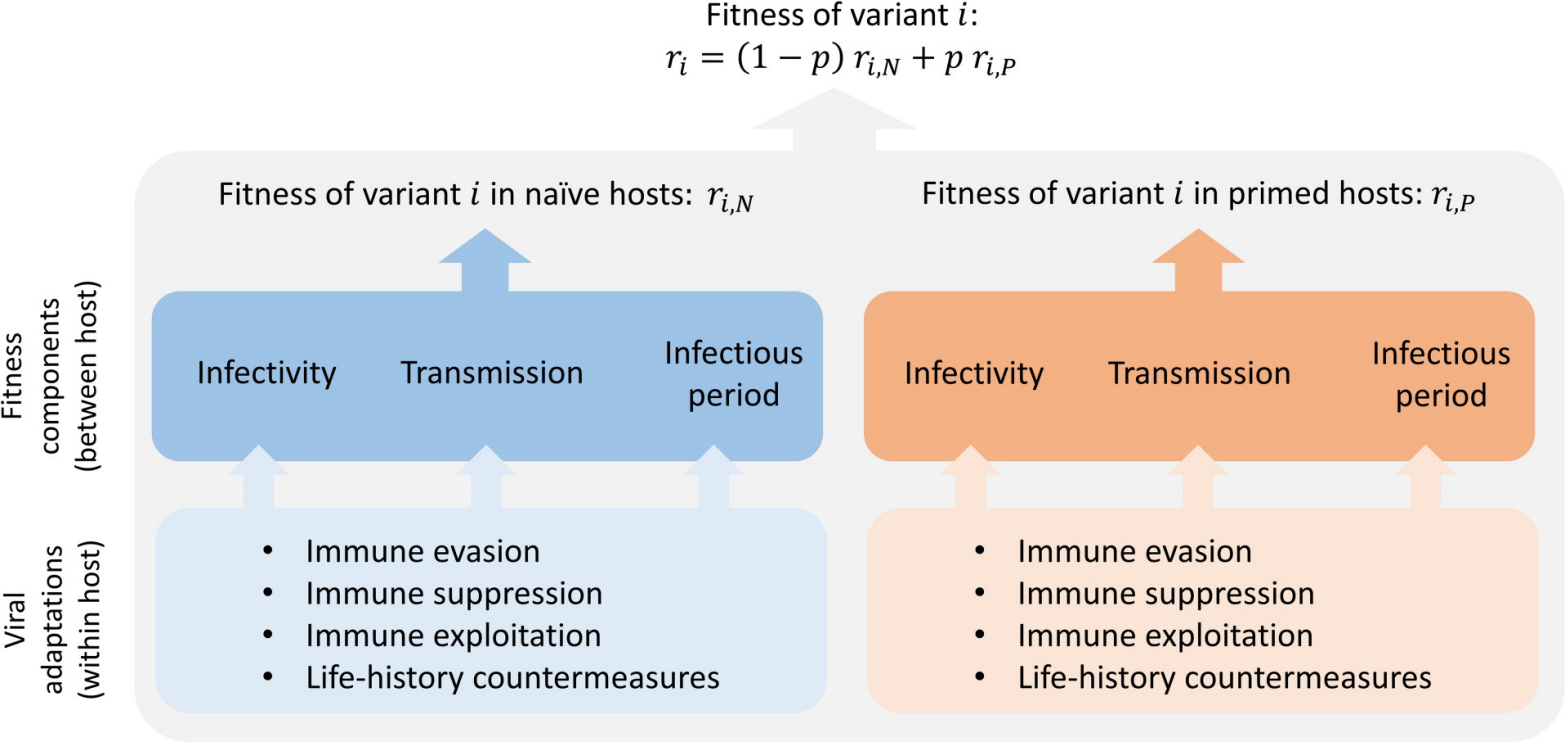
The fate of a variant (*i*) is determined by 3 key components of fitness, each of which can be affected by multiple within-host mechanisms of adaptation. All else being equal, variants with increased infectivity, increased transmissibility, or a long and early infectious period (i.e., long infections and a short generation interval) will have an increased fitness (rate of spread in a population). As indicated in Eq (1), fitness depends on both the degree of adaptation to naïve and primed hosts. Within-host processes affect the 3 components of fitness in each of the host types. Some within-host mechanisms of adaptation can be measured directly using in vitro assays. Some components of pathogen fitness can be inferred from evolutionary epidemiological studies.

With this setup, we can give a precise definition of a variant being adapted to primed or naïve host populations. If Δ*r*_*P*_>0, then the variant is more fit (i.e., has a higher growth rate) than the wild type in a population of primed hosts and so we say it is adapted to primed host populations (equivalently, it is more immunity-adapted than the wild type). Likewise, if Δ*r*_*N*_>0, then the variant is more fit (i.e., has a higher growth rate) than the wild type in a population of naïve hosts and so we say it is adapted to naïve host populations. Thus, in the first phase of an outbreak, when the fraction of primed hosts *p* is small, selection strongly favors variants for which Δ*r*_*N*_>0 whereas, in the second phase, when *p* is large, it strongly favors variants for which Δ*r*_*P*_>0. In what follows, we focus on immunity-adapted variants (i.e., those for which Δ*r*_*P*_>0). Note that while there are many molecular and cellular mechanisms within an infected host that can make a variant immunity-adapted (Box 1 and Fig 1), it is the impact of these mechanisms on the growth rate of the population of infected hosts that determines whether a variant spreads.

**Box 1. Mechanisms of adaptation.**

The ability of a variant to spread between hosts can arise from many different mechanisms operating within an infected individual. The mechanisms listed below are illustrative of the diverse range of possible within-host adaptations.

**Immune evasion (avoiding anti-pathogen responses)**

- Antigenic change
- Antigenic loss. Inactivation or deletion of molecules targeted by host responses. Examples include loss of toxins (diphtheria, pertussis)
- Antigenic repertoires. Changes in genes controlling the rates at which pathogens generate and expose novel antigens (e.g., trypanosomes, malaria)
- Increased cell–cell infection to evade antiviral humoral immunity that threatens cell-free infection [3]
- Altered tissue tropism to immune-privileged sites

**Immune suppression (dampening or misdirecting anti-pathogen responses)**

- Up-regulation of enzymes to degrade effector molecules (e.g., ptxP3 in pertussis)
- Production of immune-regulatory molecules such as cytokine mimics (e.g., pox viruses) and immune antagonists (e.g., Orf9b and Orf6 in Alpha variant of SARS-CoV-2, [4])
- Production of substances that drive inappropriate responses (e.g., helminths)
- Production of “smoke screen” molecules, which distract immune effector molecules (e.g., malaria, [5])

**Immune exploitation (utilizing host responses)**

- Antibody-dependent enhancement (e.g., [6,7])

Direct countermeasures against immunity, such as those listed above, are not the only possible within-host mechanisms that can contribute to enhanced between host fitness. A very different suite of potential mechanisms has to do with where, when, and how fast pathogens replicate.

**Other life history adaptations**

- Variants that replicate earlier or faster can overwhelm the immune response, at least initially
- Variants that replicate more slowly can potentially remain below immune detection for longer (e.g., many chronic viral infections)
- Variants that can exploit altered host cell invasion pathways can have an advantage when primary pathways are blocked by host immunity
- Variants can acquire traits that enhance fitness independent of immunity

Traits underpinning these mechanisms can include higher binding affinity to host receptors, large burst sizes (number of pathogen progeny released from a host cell), altered latency (dormancy in host cell), changes in tissue tropism, and changes in the investment of within-host replication relative to transmission stage production (e.g., malaria). Where transmission is restricted by disease severity (e.g., via host death or hospitalization), immunity (natural or vaccine induced) can enhance pathogen transmission by reducing disease severity (e.g., Marek’s disease).

**Fig 2:**
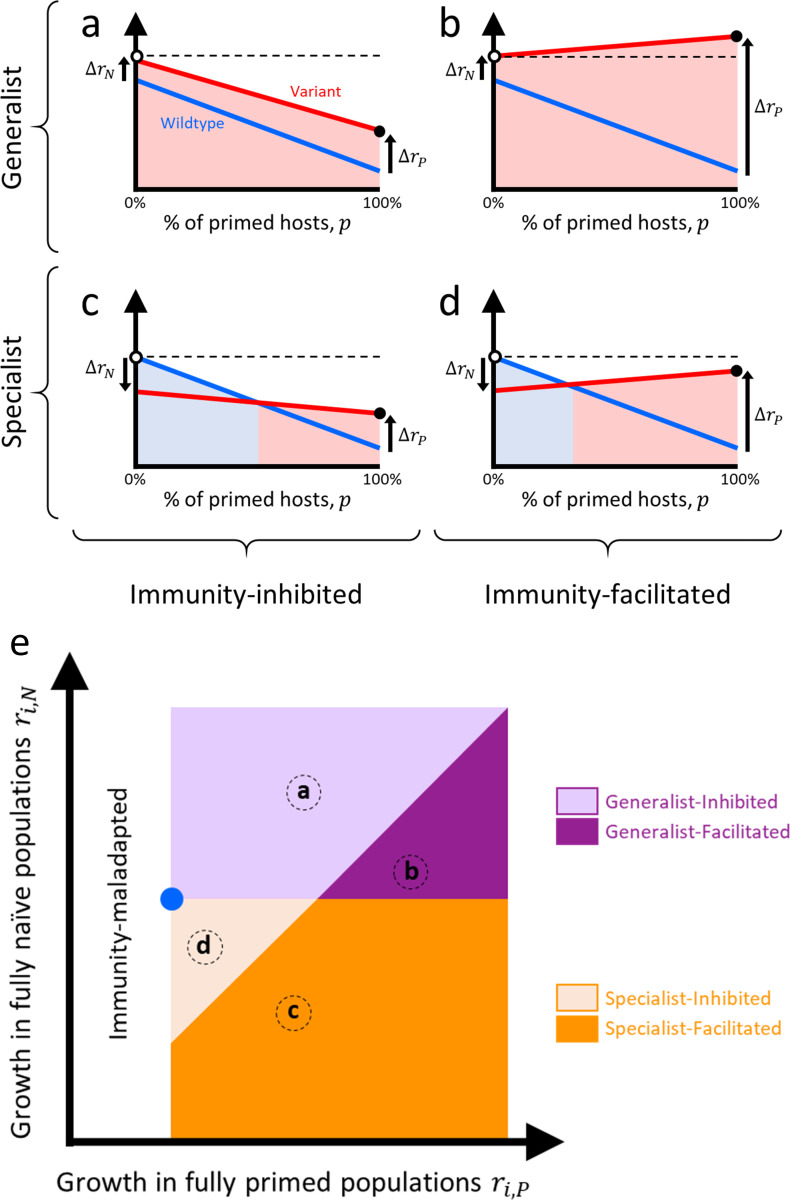
Four types of immunity-adapted variants. Solid lines depict the growth rate of the population of infected individuals for the wild type (blue) and for a variant (red) as a function of the fraction of the population that has been primed against infection by vaccination, previous infection, or both. Priming decreases the growth rate of the wild type (*r*_*N*_>*r*_*P*_). Quantities Δ*r*_*N*_ and Δ*r*_*P*_ are the differences in growth rate between the variant and the wild type in naïve and primed hosts, respectively. Colored shading indicates which type prevails evolutionarily: the wild type (light blue shading) or the variant (light red shading). Panels (**a**) and (**b**) show generalists; the variant is also better adapted to naive hosts (Δ*r*_*N*_>0). Generalist variants will outcompete the wild type even in the absence of priming. Panels (**c**) and (**d**) show specialists; the variant is maladapted to naïve hosts (Δ*r*_*N*_<0). Specialist variants will outcompete the wild type only above a critical threshold. Panels (**a**) and (**c**) show immunity-inhibited variants; the growth rate of the variant decreases with increasing fractions of primed hosts. As a result, the growth rate of infections after adaptation (i.e., after fixation of the fittest type) in a fully primed population (black dot) is always lower than that in a fully naïve population (white dot and dashed line). Panels (**b**) and (**d**) are immunity-facilitated variants; the growth rate of the variant increases with increasing fractions of primed hosts. As a result, the growth rate of infections after adaptation in a fully primed population (black dot) is always higher than that in a fully naïve population (white dot) for generalist variants (panel (**c**)) but it can go either way for specialists (panel (**d**); only the case where it is lower is shown). Panel (**e**) show a plot of the growth rate of variants in a fully naïve (*r*_*i*,*N*_) and a fully primed (*r*_*i*,*P*_) population. Blue dot indicates location of the wild type. Uncolored region corresponds to variants whose growth rate in primed hosts is less than that of the wild type and so are immunity-maladapted (and so ignored in our discussion). Different colored regions correspond to the 4 types of variants from panels (**a–d**). Finer distinctions within these types are presented in S1 Fig. See S2 Appendix for a discussion of alternative ways to visualize variants.

The above ideas lead to 2 useful ways of categorizing immunity-adapted variants. First, if an immunity-adapted variant is also adapted to naïve host populations (i.e., Δ*r*_*N*_>0), then we refer to it as a “generalist” variant since it is better at spreading than the wild type, irrespective of host type. Conversely, if an immunity-adapted variant is maladapted to naïve host populations (i.e., Δ*r*_*N*_<0), then we refer to it as a “specialist” variant since it is specialized to have higher fitness than the wild type in primed host populations only. This categorization is useful because, for immunity-adapted variants, generalists will increase in frequency and replace the wild type regardless of the fraction of the population primed, whereas specialists require the fraction primed to be above a critical threshold before they will increase in frequency (Fig 2), such as after a vaccination campaign.

A second useful way to categorize a variant is to assess whether the absolute growth rate of infections that it causes is inhibited or facilitated by immune priming. The absolute growth rate of an immunity-inhibited variant decreases as the fraction of the population primed increases, whereas the absolute growth rate of an immunity-facilitated variant increases with increased priming (Fig 2). This categorization is useful because it speaks to whether the spread of infection will ultimately be lower or higher because of priming and subsequent immunity-driven pathogen evolution. If a variant’s growth rate is immunity-inhibited, then increasing the fraction of primed hosts will always reduce the overall spread of infection, even if the variant ultimately replaces the wild type (Fig 2A and 2C). However, if a variant’s growth rate is immunity-facilitated, then if priming drives the variant to replace the wild type, it is possible that the overall spread of infection goes up (Fig 2B).

The categorization of variants in Fig 2 is based on their per capita growth rates, and such plots are therefore specific to immunological context. For example, the same variant might be categorized differently in populations that differ in the type of vaccination, the recency of vaccination, or the proportion of priming that is due to vaccination versus recent infection. Likewise, such plots are specific to epidemiological context as well. For example, early in an outbreak there is typically exponential growth in the number of infections, but as an outbreak progresses and/or non-pharmaceutical interventions are introduced, the force of infection will eventually decline, reducing all growth rates (*r*_*i*,*N*_ and *r*_*i*,*P*_). Eventually, if the pathogen becomes endemic, the average growth rate across all variants will be zero. Notice, however, that the categorization of variants in Fig 2 depends only on the relative growth rates, and so the very same framework can be applied to any immunological or epidemiological context (e.g., in the early stages of an outbreak during exponential growth or at equilibrium once the pathogen is endemic). Moreover, if the relative ordering of variants does not change with context, then their classification into 1 of the 4 categories will remain consistent regardless of what is happening epidemiologically (S1 Appendix).

**Fig 3:**
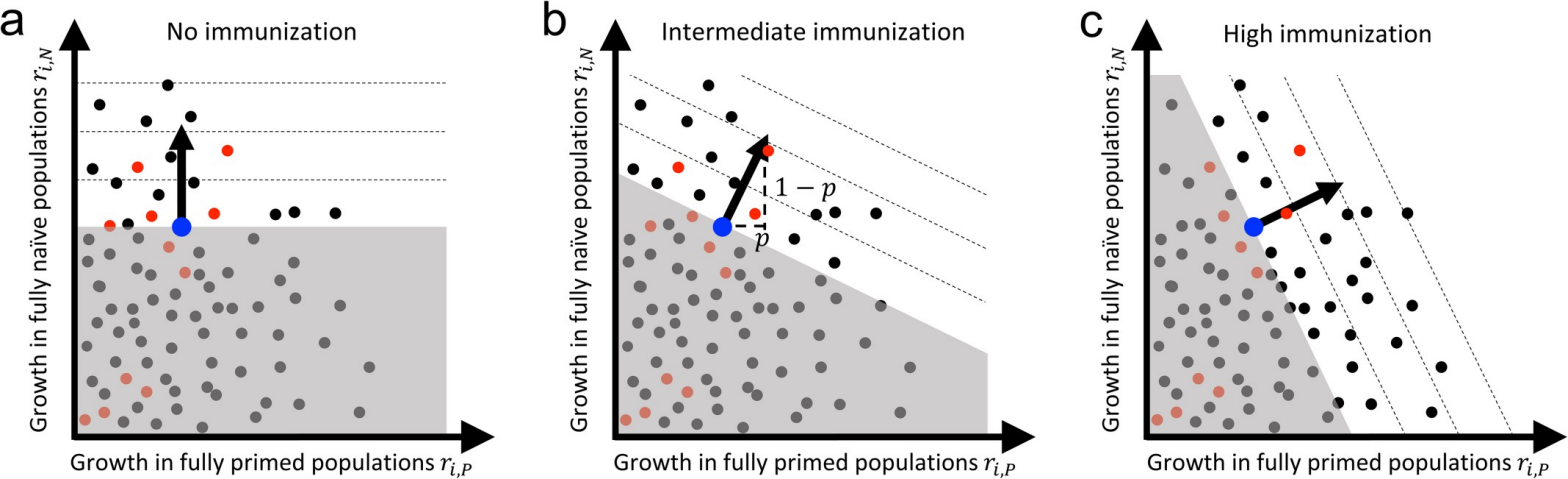
Selection and genetic variation. Plots of the growth rate of all viable variants (*i*) in a fully naïve and a fully primed population (black dots). Large blue dot denotes the current wild type. Red dots are those variants that are most accessible from the wild type. Note that the location of all variants along the *r*_*i*,*P*_ axis is specific to an immune response and may be different for natural immunity and different vaccines. All variants in the white region are selectively advantageous but variants in the direction of the selection arrow are most strongly favored (dashed lines indicate contours of overall growth rate). Variants in the gray region are disfavored by selection. The direction of selection arrow is upwards in a fully naïve population (*p* = 0) (panel (**a**)) and shifts towards the right as the fraction of primed individuals increases (panels (**b** and **c**)).

To conceptualize evolutionary change as the fraction of primed hosts increases (such as during a vaccination campaign), we can then construct a plot of the absolute growth rate of different possible variants in each host type, locating on the plot each of the 4 types of variants from Fig 2 (alternative ways of plotting variants are discussed in S2 Appendix). We can also use such a plot to illustrate how the nature of selection changes as the fraction of primed hosts increases (Fig 3). In Phase 1, when most hosts are naïve (i.e., *p* is small), selection will primarily favor variants with a larger growth rate in naïve hosts (Fig 3A). As we move to Phase 2 (Fig 3B), however, an increasing fraction of hosts are primed (i.e., *p* increases) and selection shifts to primarily favoring variants with a larger growth rate in primed hosts (Fig 3C). Throughout this transition, the variants that appear can be specialists or generalists and either immunity-inhibited or immunity-facilitated.

**Fig 4:**
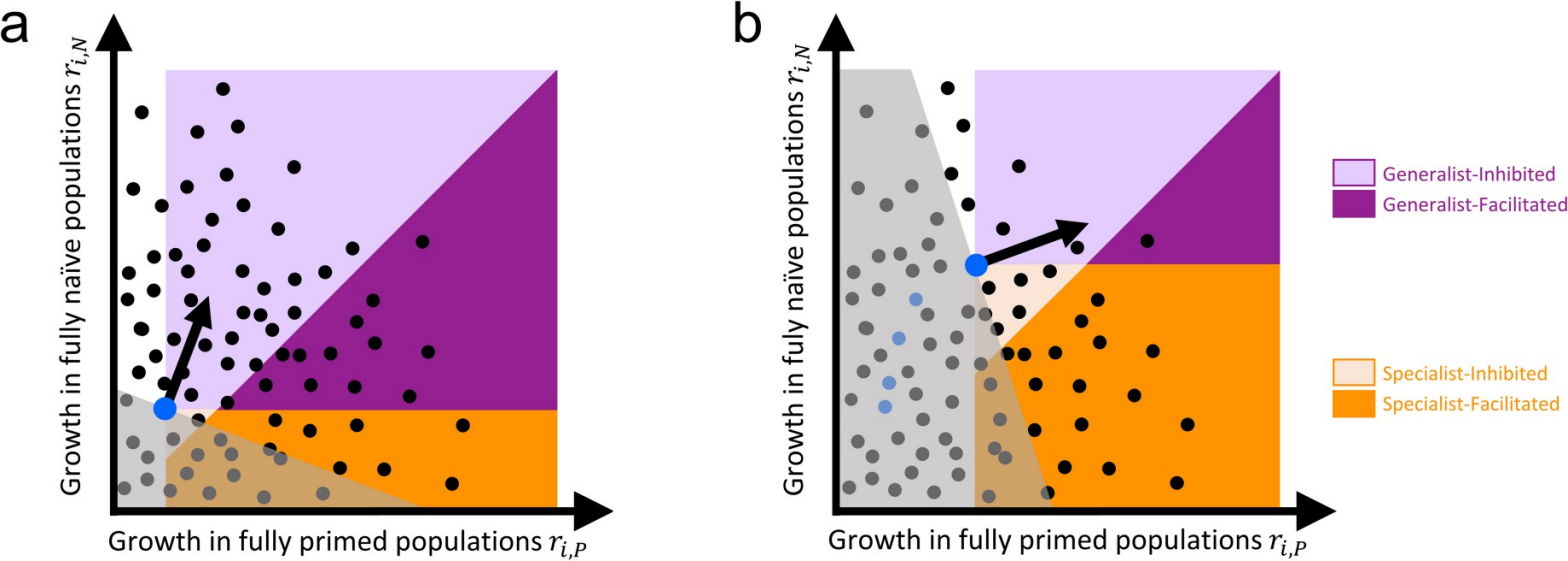
Pathogen adaptation as the fraction of primed individuals increases. Plots of the growth rate of all viable variants in a fully naïve and a fully primed population (dots). Large blue dot denotes the phenotype of the current wild type and black arrow indicates direction of selection (i.e., the variants that are most advantageous). Variants in the gray region are disadvantageous. Note that the location of all variants along the *r*_*i*,*P*_ axis is specific to immune response and may be different for natural immunity and different vaccines. Colored regions indicate the 4 different kinds of variants. (**a**) Early in a novel host–pathogen association when a small fraction of hosts are primed. Many potential new variants will be better adapted to both host types (i.e., they will be generalists). (**b**) Later in the association, when the pathogen is better adapted to its novel host (and a larger fraction of hosts are primed). The evolutionary trajectory of successive fixation events leading to the new wild type variant is indicated by the succession of blue dots. Note how the change in the location of the blue dot can affect the typology of some variants (i.e., a variant that was identified as a generalist in the early stage of adaption could later become a specialist relative to the more recent form of the pathogen). Once the level of adaptation is high (panel (**b**)), most advantageous variants that appear will tend to be specialists. Even though generalists are still more strongly favored by selection there are fewer of them that can arise.

With this framework, evolutionary theory then makes some predictions about how we expect adaptation in novel pathogens to unfold as population priming increases (such as during a vaccination campaign). As a pathogen adapts, there will be occasional selective sweeps in which a new variant displaces the wild type and becomes the new wild type. The sequence of selective sweeps that occurs will be determined by both the direction of selection (the arrow in Fig 3) and the set of variants that happen to appear (Box 2). Initially, in a new host–pathogen association (such as humans and SARS-CoV-2), there will typically be abundant scope for adaptation to both naïve and primed hosts, and thus a great many of the variants that arise and become dominant will be generalist variants (Fig 4A). Over time, as the pathogen becomes better adapted to the novel host, and as the fraction of primed hosts increases, there will be fewer new variants that increase fitness in both host types, leaving primarily specialist variants as the source of variation for further adaptation (Fig 4B). Thus, as a pathogen becomes increasingly adapted to a novel host, adaptation to primed hosts will tend to result in the loss of some degree of adaptation to naïve hosts.

**Box 2. Evolutionary steps leading to adaptation to host immunity.**

Pathogen adaptation requires variation in fitness among variants. New variants arise from mutation during replication and from recombination when distinct variants coinfect the same host. It is important to distinguish between the rate at which new variants arise and how their fitness differs from the wildtype.

**The rate at which variants arise**

Mutations are continuously generated during the replication of the pathogen within infected hosts. The rate at which this occurs is proportional to the rate at which genomic changes occur during replication, and the amount of replication that is taking place. Immunity (natural or vaccine induced) reduces the amount of replication in 2 ways. First, at the within-host level, if a primed host is infected, the immune response is expected to reduce the pathogen load and to clear the infection faster. Second, at the between-host level, a high fraction of primed individuals in the population is expected to reduce the number of infected hosts (both naïve and primed). However, these effects are tempered for imperfect (or leaky) vaccines because they have a lower ability to reduce pathogen replication and to prevent infection.

**The fitness effects of variants**

The fate of a new variant is determined by how the rate of change of the number of infections it causes differs from that of the wild type in both naïve and primed populations (i.e., where it falls in Fig 3 relative to the wild type). To this end, it is useful to distinguish between the set of variants that are possible (all the dots in Fig 3) and the set of variants that are readily accessible genetically from the wild type (the subset of red dots in Fig 3). There will be biological constraints on the magnitude of growth rate that is possible in the 2 host types and therefore all the dots in Fig 3 will fall within some specific region of the plane. Most mutations are expected to be deleterious or have little effect, but some may result in a larger growth rate than the wild type [8,9]. Hence, we expect a high density of possible phenotypes (black dots in Fig 3) with low fitness relative to the density of phenotypes that increase fitness in both host types. Within this set of possible variants, some will be more readily accessible from the current wild type than others for several reasons. First, some variants might be multiple mutational or recombinational steps away from the wild type and so will be exceedingly unlikely to arise. For example, the lack of adaptation of measles virus to vaccines despite decades of global vaccination is potentially because variants that can escape a polyclonal antibody response require at least 5 new mutations to the H glycoprotein [10]. Second, competition between the variant and the wild type within an infection can promote (or hamper) the variant’s ability to reach a density high enough for onward transmission to occur. For example, in novel host–pathogen associations, mutations that are beneficial for within-host competition are also likely to be beneficial in other respects, including their ability to spread at the between-host level simply because more generalist variants are accessible when the wild type is poorly adapted to its host. As the association becomes more established, however, variants that are successful within hosts will tend to have reduced success at the between-host level. This effect of within-host selection biasing the set of variants that are accessible to between-host selection is likely also modulated by the strength of immunity (e.g., the leakiness of a vaccine [11]).

**Vaccination and the speed of pathogen adaptation**

Faster rollout and more effective vaccines will, all else being equal, limit the emergence of new variants. Hence, the use of “leaky” vaccines (i.e., vaccines that do not completely prevent infection and onward transmission) and the occurrence of chronic infections in immunocompromised hosts could speed up pathogen adaptation both because they increase the flux of mutation relative to the use of non-leaky vaccines and because they facilitate the within-host rise of some immunity-adapted variants. Once an immunity-adapted variant is circulating in the population, the influence on evolutionary adaptation of the rate at which it arises through mutation is negligible compared to the selection acting on the variant (e.g., the dynamics of the Alpha and Delta variants of SARS-CoV-2 were driven by selection, not by the flux of mutations [12,13]). In this case, the speed of pathogen adaptation is mainly driven by selection and different targeted vaccination strategies may provide ways to slow down this adaptation [14–16].

It is more difficult to make predictions about whether variants are likely to be immunity-inhibited or immunity-facilitated. At first, one might wonder if immunity-facilitated variants are even possible but, as we detail in the next section, such variants have been documented in some infectious diseases. Moreover, it is not difficult to imagine how such a variant might occur for SARS-CoV-2. For example, people with symptoms often isolate and socially distance so they do not infect others. A variant that evades immunity in terms of its transmissibility could spread more rapidly in a fully primed population than in a fully naïve population (i.e., it would be immunity-facilitated) if priming reduces disease severity and so reduces the rate of isolation and social distancing. This hypothetical example helps to emphasize that categorizing a variant as immunity-facilitated is solely a statement about its fitness (i.e., its ability to spread) and it carries with it no a priori implication about whether the spread of such a variant would ultimately lead to a greater or lesser amount of disease, either in an individual infection or in the population overall.

The above theory is general and applies to both infection-primed and vaccine-primed hosts. The immunity generated by these 2 methods may well be similar, but it need not be. In the rest of this Essay, we explore the above theory in the context of vaccine-primed hosts specifically.

## Examples of pathogen adaptation to vaccination

Before considering examples of adaptation to vaccine-primed hosts, or equivalently “vaccinated hosts,” it is important to stress that many vaccines have not been undermined by pathogen adaptation (e.g., smallpox, measles, polio). This lack of adaptation is hypothesized to result from 2 features commonly associated with vaccination [17]. First, because vaccination is a prophylactic intervention, it can keep pathogen numbers small within vaccinated hosts, which limits the generation and transmission of novel variants. Second, because vaccines typically induce immune responses against multiple targets on a pathogen, multiple genetic changes may be required to circumvent vaccine-mediated immunity [10]. Both features are expected to limit the ability of the pathogens to adapt to vaccination by hampering the accessibility of variants (fewer red dots in Fig 3 and Box 2). However, for a handful of vaccines that do not keep pathogen densities below transmissible levels in the majority of infected hosts, or that do not induce immunity against multiple targets, evolutionary adaptation has occurred [17]. Given these caveats, we look to these previous examples for guidance on possible outcomes of adaptation to vaccination in SARS-CoV-2.

The most direct way to determine how vaccines affect pathogen adaptation is through experimental evolution, yet we know of only one study that takes this approach. It involved a novel host–pathogen association of malaria parasites with laboratory mice [18]. Parasites were serially passaged for 20 generations through either vaccinated or naïve mice and allowed to evolve in response to these different treatments. The parasites became progressively better able to replicate in the host type they were evolving in, but they also evolved a better replication rate in the other host type as well. Moreover, vaccination inhibited the replication of all the evolved pathogens, demonstrating that the variants that arose during evolution were immunity-inhibited generalists.

Most other data are observational and focus on pathogen species that have a long-term association with their host. As expected from the earlier considerations, many immunity-adapted variants appear to be specialist variants relative to the wild type. For example, immunity-adapted variants of hepatitis B virus that arise following vaccination have altered surface antigens, making the vaccine less effective [19]. These variants cause sporadic breakthrough infections but they have not increased in overall number at the population level even as vaccination rates have increased [20,21]. This suggests that, although they are more fit than the wild type within vaccinated hosts, their spread from vaccinated hosts is apparently suppressed, making them immunity-inhibited specialists. For *Bordetella pertussis*, the use of acellular vaccines that target pertactin have led to the spread of immunity-adapted variants that no longer express pertactin [22]. These variants appear to be more fit than the wild type in vaccinated hosts but less fit in naïve hosts, making them specialist variants [23]. Variants also arise that overexpress the immunosuppressive pertussis toxin molecule, and these appear to be more fit than non-overexpressing variants in both naïve and acellular-vaccinated hosts [24]. Notably, fitness was not assayed in hosts vaccinated with whole-cell vaccines, limiting our ability to definitively classify the variants as specialists or generalists. For both sets of *B*. *pertussis* variants, however, the ability of the variants to spread in a vaccinated population appears to be less than in naïve populations [23,24], making them all immunity-inhibited variants.

Similar patterns often arise with vaccines used in farm animals, although the data necessary to distinguish between specialist and generalist variants are often inconclusive. For example, avian metapneumovirus vaccination suppresses virus shedding in turkeys, but less so for recent isolates of the virus than for historical isolates, and no difference was detected between the isolates in non-vaccinated turkeys [25]. This difference has been credited to amino acid coding divergence in 2 genes [25]. Similarly, breakthrough against a vaccine for the fish bacterial pathogen *Yersinia ruckeri* is associated with a loss of the bacterial flagellum [26]. However, partial vaccine protection persists against all tested variants [27], again suggesting that these variants are immunity-inhibited.

One strikingly different example is the chicken pathogen Marek’s disease virus (MDV). MDV is an oncogenic virus that can cause paralysis and high levels of mortality [28], and a succession of vaccines have been developed and deployed in response to continual vaccine-driven evolution [29]. The immunity-adapted variants that have been analyzed appear to be disfavored in naïve chickens relative to the ancestral virus [30]. Nevertheless, unlike the examples described above, the immunity-adapted variants of MDV transmit better from vaccinated chickens than from naïve chickens [30]. These variants are therefore examples of immunity-facilitated specialist variants. Notably, the overall prevalence of disease in the poultry industry was nevertheless reduced by vaccination despite this evolution [31] (as in Fig 2D).

Other examples of evolution in response to vaccination involve host–pathogen associations in which multiple serotypes coexist and vaccines target only a subset of those serotypes. These situations are more complex because the very coexistence of serotypes suggests that multiple host types are present, possibly because of distinct immunological histories that have arisen through natural infection by the different serotypes. As a result, the framework in Figs 3 and 4 would need to be extended with additional axes corresponding to the different kinds of hosts, since vaccination and natural infection appear to prime hosts in different ways in this system. Nevertheless, we can draw an analogy to the previous examples by viewing the set of serotypes targeted by the vaccine as the “wild type” and the non-targeted serotypes as the “variants.” The fact that the wild type and variant serotypes coexist suggests that, as expected, they are specialist variants. It is more difficult to categorize them as being immunity-inhibited or immunity-facilitated, but in all examples that we are aware of, the total prevalence of infection has either gone down or remained unchanged after the deployment of the vaccine. For example, vaccination against *Streptococcus pneumoniae* often resulted in no change in the total prevalence of bacterial carriage because non-targeted serotypes completely replaced vaccine-targeted serotypes following vaccination (although disease burden has been reduced) [32–34]. By contrast, for human papillomavirus, vaccination reduced the total number of infections because non-targeted serotypes did not change in prevalence while vaccine-targeted serotypes became less common [35]. Other examples involving coexisting serotypes, including *B*. *pertussis* [36], *Haemophilus influenzae* [37], *Neisseria meningitidis* [38], and rotavirus [39], appear to fall somewhere between these 2 extremes.

One final example is human influenza virus, which continually evolves in response to host immunity through a process known as antigenic drift, generating many sequential influenza variants over time [40]. To keep up with antigenic drift, flu vaccines are frequently updated. Again, this can be conceptualized in the current framework by introducing a new axis in Figs 3 and 4 every time a new vaccine is introduced and/or a new immunological type of host arises. We were unable to find definitive data that addresses whether influenza variants tend to be generalists or specialists. Either way, existing data suggest that most novel variants arising through antigenic drift are partially inhibited by vaccination, making them immunity-inhibited variants [41].

Thus, in the handful of cases where vaccine adaptation has been observed, specialist variants have been involved. This is consistent with our theoretical expectation that generalist variants will eventually give way to specialist variants as novel host–pathogen associations become more established (Fig 4). Moreover, most of those handful of cases involve immunity-inhibited specialists. As a result, vaccination has generally resulted in a reduced overall spread of infection, even when vaccination drove the evolutionary advantage of the variants. We have identified examples of immunity-facilitated specialist variants, but it is noteworthy that even in these cases, it appears that such a vaccine-driven increase in the overall prevalence of disease has never been documented [42].

We are unaware of any examples of immunity-facilitated generalist variants in any infectious disease. Such a variant would spread regardless of vaccine coverage, and it would also necessarily compromise our ability to control infection using that particular vaccine (as in Fig 2C). It is not clear if the apparent absence of such variants is because very few variants in this category are possible (Box 2), or if it is because generalist variants will be rare, except when host–pathogen associations are new. As discussed above, it is possible to imagine such variants, but again we stress that even if they arose, their spread need not necessarily lead to a greater overall amount of disease in either infected individuals or at the population level.

### SARS-CoV-2

There is now substantial evidence that SARS-CoV-2 has been undergoing rapid adaptive evolution since its first appearance in humans. The first compelling data involved the spread of the Alpha and Delta variants because of their fitness advantages over the wild type [12,13,43]. What does our framework tell us about the potential for SARS-CoV-2 adaptation to primed hosts? Epidemiological data from several countries suggest that, as expected, the main immunity-adapted variants to appear initially were immunity-inhibited generalists. The Delta variant increased in frequency in countries with very low vaccine coverage, as well as in countries with relatively high vaccination coverage, suggesting that it was a generalist. Data indicating that Delta was immunity-inhibited are less direct and come both from epidemiological studies [44] and from neutralization assays [45]. Although these data only quantify 1 of the 3 components of fitness (see below section on the relationship between pathogen fitness and infection characteristics), the BNT162b2 Pfizer-BioNTech, mRNA-1273 Moderna, and ChAdOx1 nCoV-19 Oxford-AstraZeneca vaccines nevertheless still provided protection against infection [46,47]. The case for the Alpha variant being immunity-adapted is even less direct because Alpha spread and was then largely replaced by Delta before significant vaccine coverage or natural immunity existed in most countries. Thus, the epidemiological data clearly show that Alpha was advantageous relative to the wild type in naïve hosts [12,48,49], but estimates of its fitness in primed hosts again come from proxies using vaccine efficacy. The important point for both variants is that they would have become dominant regardless of whether vaccines had been deployed because they are generalists.

More recently, Omicron variants have spread widely, replacing the Delta variant everywhere [50]. Omicron variants appear to be immunity-adapted [51–53] and preliminary observations suggest that they are immunity-inhibited [54,55]. At this stage, however, it is not clear if Omicron variants are generalists or specialists. Part of the reason for uncertainty is that vaccine coverage and priming through natural infection is now reaching high enough levels in many countries that it has become more difficult to assess the fitness of variants in naïve hosts. At the time of writing (July 2022), new Omicron sub-lineages continue to arise and spread, suggesting that further viral adaptation is likely. As mentioned above, vaccine-driven evolution has tended to occur in other pathogens when either the benefits of prophylaxis are small (e.g., the vaccine does not sufficiently suppress pathogen replication below transmissible levels) or when they target a small number of pathogen epitopes [17,56]. Data increasingly suggest that at least the first of these is true for SARS-CoV-2 and currently deployed vaccines [57–60]. As SARS-CoV-2 adapts further to humans, we might therefore expect that specialist variants will begin to appear that have even higher reproductive success in primed populations but where this increased adaptation to the primed hosts comes at a cost of reduced reproductive success in naïve populations.

As far as we know, immunity-facilitated variants of SARS-CoV-2 have not yet been reported and, depending on the available genetic variation (Box 2), it is possible that they never will arise. For a variant to be immunity-facilitated, immunity would have to either increase the rate at which the variant generates new infections and/or decrease the rate at which existing infections caused by the variant are lost from circulation through recovery, isolation, or death. In theory, molecular processes involving antibody-dependent enhancement (ADE) of cell infectivity could provide a mechanism by which immunity facilitation occurs [6,7,45], but we know of no evidence that ADE has increased transmission in any infectious disease. Immunity could also potentially increase the rate at which a variant generates new infections if primed people engage in more risky behavior (e.g., vaccinated people are allowed entry to concerts and bars [61]). The other type of variants that could theoretically be facilitated by immunity are variants whose transmission is curtailed because they cause more severe disease (e.g., leading to isolation). Vaccination, which is aimed at reducing disease severity, could also potentially facilitate the silent or semi-silent spread of such variants (Box 2) in a manner directly analogous to the variants facilitated by the first-generation vaccines against Marek’s disease [30].

In the longer term, if variants like those hypothesized above appear and spread, thereby compromising the utility of current vaccines, it is likely that boosters and new vaccines would be introduced. Regardless of whether such variants appear, as SARS-CoV-2 spreads in the human population and presumably becomes an endemic virus, the number of people with an immunological history due to natural infection will increase substantially. As a result, the framework presented here will need to be extended to account for multiple host types. Making longer-term predictions for such cases is difficult at this stage because a great deal will depend on the nature of the genetic variation that is possible (Box 2).

## The relationship between pathogen fitness and infection characteristics

The above analysis focuses solely on pathogen fitness. One thing missing from this discussion is a consideration of how vaccination and natural immunity might drive the evolution of infection characteristics such as vaccine efficacy or disease severity. To better illustrate the relationship between the fitness of a variant (as measured by the growth rate of infections that it causes) and the characteristics of the infection, we can decompose the absolute growth rate (*r*_*i*_) of a variant into 3 main components of fitness (Fig 1 and Box 1): infectivity (the probability that, upon exposure, a variant infects either type of host), transmissibility (the rate at which a variant produces infectious propagules from either host type that contact uninfected individuals), and the infectious period (the time period during an infection in each host type when a variant produces infectious propagules). All else being equal, variants with increased infectivity, increased transmissibility, or a long and early infectious period (i.e., long-lasting infections and a short generation interval) will have an increased growth rate.

### Vaccine efficacy against infection

The infectivity of a variant is a key property for determining how well a vaccine works against a variant. If *σ*_*N*_ and *σ*_*P*_ denote the infectivity of a variant in naïve and (vaccine) primed hosts respectively, then vaccine efficacy (*VE*) is the proportional reduction in infectivity that vaccination confers, given by *VE* = 1−*σ*_*P*_/*σ*_*N*_. This highlights 2 important things about the utility of *VE* for understanding the evolutionary epidemiology of immunity-adapted variants. First, because *VE* is a measure of the relative infectivity of a variant in vaccinated versus non-vaccinated hosts, a variant can have a reduced *VE* as a result of an increase in *σ*_*P*_ and/or a decrease in *σ*_*N*_. Second, *VE* involves only 1 of the 3 different components of fitness and so it provides only partial information for determining the fate of a variant or the consequences it will have if it sweeps to fixation. For example, the Beta and Gamma variants of SARS-CoV-2 both appear to have a reduced *VE* [62] yet, to date, neither has become the dominant variant. Measures of *VE* that capture other components of pathogen adaptation to vaccinated hosts do exist [63].

A related issue arises in discussions of vaccination that center around so-called “escape variants.” Although this term is not always defined precisely, it is often used in reference to variants that differ in epitope and so are able to escape a specific immune response as measured in inhibition assays in vitro [3,62,64–66]. For example, SARS-CoV-2 variants are sometimes characterized by both their transmissibility (as measured by their overall growth rate and/or *R*_0_) and their performance in inhibition assays. We have purposefully avoided using this type of characterization here because this approach conflates the mechanism through which a variant is potentially adapted to primed hosts (i.e., escape from a specific immunity and so greater ability to replicate within an individual) with the source of selection that favors the variant (e.g., increased infectivity). It is useful to keep these notions distinct because there are many different mechanisms through which a variant can be adapted to primed hosts (Box 1) and each of these can affect any of the 3 main epidemiological components of fitness (i.e., infectivity, transmissibility, and infectious period; Fig 1). Therefore, we believe the most consistent, general, and agnostic way to characterize variants is as described in Fig 3. Ideally, we would also quantify several distinct infection characteristics (infectivity, transmissibility, and infectious period) for variants that arise, along with this quantification of fitness (S1 Appendix). Such an approach is possible for SARS-CoV-2 using the unprecedented availability of genetically resolved, real time epidemiological data (Box 3).

**Box 3. How to characterize the fitness of SARS-CoV-2 variants?**

The ongoing SARS-CoV-2 pandemic is characterized by an unprecedented access to incidence and sequencing data in real time. This data provides a unique opportunity for quantifying the underlying components of pathogen fitness (infectivity, transmissibility, and infection duration) related to adaptation to naïve and primed hosts. Three main dynamical variables carry useful information about these components of fitness (S1 Appendix).

First, the per capita growth rate of the epidemic provides information about the potential emergence and the spread of new variants. Any deviation from the predicted drop in incidence of the wild type due to the build up of natural immunity and increasing vaccination coverage could signal the spread of an immunity-adapted variant (Δ*r*_*P*_>0).

Second, analysis of the change in frequency of a variant allows some inference to be made about which components of fitness underly adaptation to natural immunity or vaccination. We show in S1 Appendix that the magnitude of change in the frequency of a variant will be proportional to the availability of susceptible hosts and the proportion of primed hosts if the variant obtains its advantage through increased transmissibility (*β*) or infectivity (*σ*), but this change will be independent of susceptible hosts if the variant obtains its advantage through a longer infection duration. Therefore, as the availability to susceptible hosts varies with lockdowns and other non-pharmaceutical interventions, as well as with the coverage of vaccination, tracking how this affects the change in variant frequency can inform us about the mechanism underlying the variant’s success [67,68].

Third, the overrepresentation of a variant in primed hosts can be used as an early signal that the variant is immunity-adapted. We show in S1 Appendix that the difference in variant frequency between naïve and primed hosts (i.e., the genetic differentiation of the pathogen populations in the 2 types of hosts) is mainly governed by the relative infectivity of the variant in primed hosts, but not by its transmissibility. Hence, the analysis of these 3 dynamic variables provides a way to begin disentangling the 3 major components of fitness.

### Disease severity

Arguably, the most important infection characteristic from the standpoint of human health is the severity of disease caused by a variant. Most definitions of severity capture both the morbidity and the mortality caused by infection. As such, severity can affect all 3 components of fitness. For example, high disease severity might reduce infection duration through increased mortality, or it might reduce the transmissibility through a reduction in activity level and thus the contact rate of infected individuals [69]. In most cases, disease severity per se is disadvantageous to the pathogen and thus selected against [70]. It is nevertheless difficult to make predictions about how disease severity will evolve because variants that cause more severe disease might have increased fitness relative to the wild type through differences in other components of fitness [67]. For example, data suggests that the Alpha variant of SARS-CoV-2 may cause more severe disease than the Wuhan wild type [71,72], but it nevertheless has higher fitness because its transmissibility is higher. In addition, disease severity may be partially mediated by the host immune response, and some in vitro studies suggest that certain antibodies may “enhance” the replication of the virus and induce more symptoms [6,73]. A SARS-CoV-2 variant that could escape from neutralizing antibodies and exploit this enhancing effect could theoretically lead to greater disease severity in primed hosts [7]. This illustrates that, although we can make quite robust and reliable predictions about the evolution of pathogen fitness in naïve and primed hosts, it is harder to make predictions about the underlying components of fitness or disease severity since variants with very different values of the 3 fitness components can nevertheless have the same overall fitness (Boxes 1 and 3). This means that pattens of evolution in these infection characteristics are likely to be somewhat idiosyncratic. This is a major reason why we cannot extrapolate the evolutionary trajectories of such traits from one pathogen to another.

Despite the lack of robust theoretical predictions about disease severity, a few observations from other infectious diseases could be relevant to SARS-CoV-2. First, vaccine protection tends to be even more evolutionarily robust against disease than against infection. This conclusion arises from the observation that when pathogens have evolved in response to vaccines in the past, vaccinated individuals that are infected by a pathogen tend to have better outcomes than non-vaccinated individuals [42]. A potential concern is if there are enhancing effects of antibodies on disease severity [74,75], as there could be for COVID [6,7,73]. Second, for pathogens with coexisting serotypes, vaccine-driven serotype replacement could in principle increase or decrease overall disease burdens if different serotypes have different propensities for causing disease, as they often do (for example, [76]). Rational design of variant-based vaccines must therefore consider both the current prevalence of each variant and their likelihood of causing disease following infection. Third, under certain conditions, vaccines may lead to the evolution of highly virulent variants. The best example of this is MDV, in which highly virulent variants of the virus kill their hosts so quickly that they are unable to persist in the absence of vaccination [30]. Vaccines ameliorate the disease severity of MDV and therefore allow hosts infected by these highly virulent variants to remain alive, but they do not prevent transmission. However, despite this effect, vaccinated chickens exposed to these highly virulent variants are nevertheless better off than non-vaccinated chickens exposed to the original wild type. By contrast, non-vaccinated chickens are now at greater risk of infection with variants causing more severe Marek’s disease than they were prior to the introduction of the vaccine. Regardless of whether SARS-CoV-2 follows this path, vaccination remains our most effective tool to mitigate the epidemic, as was the case with MDV [31]. Vaccination also reduces the number of cases, which may also slow down the flux of new mutations and thus the probability of pathogen adaptation (Box 2).

## Implications for SARS-CoV-2

If further adaptation of SARS-CoV-2 occurs in response to immune priming, then our framework and the examination of previous experimental and empirical examples suggest that the long-term outcome will likely yield specialist variants. The path to getting there will likely involve immunity-inhibited variants, meaning that we are likely to, at least partially, retain the benefits of vaccination with first-generation SARS-CoV-2 vaccines in the short term. In the meantime, there is an urgent need to monitor the epidemiology and evolution of the virus [56]. This will better characterize newly arising variants (Box 3) and make it possible to decide if, like for influenza, new vaccines are needed to counteract viral adaptation.

It is also critical to stress that concerns about possible future viral evolution are not a reason to withhold currently available vaccines. First, vaccines are currently greatly reducing disease burdens and saving lives [77]. Second, as discussed above, much of the evolution that has occurred in SARS-CoV-2 involves generalist variants that would have spread even had existing vaccines been withheld. Third, immunity arising from natural infections will also impact on-going viral evolution. It is impossible to know a priori whether natural immunity or vaccine-induced immunity will be the stronger evolutionary driver. Fourth, even with the more recent variants of SARS-CoV-2, current mRNA vaccines substantially reduce the probability of infection and infection duration compared to infections in naïve individuals [54,55,59,60,62], which very substantially reduces evolutionary potential (Box 2).

Going forward, it is quite possible that new vaccine schedules (e.g., higher doses, boosters, combinations of existing vaccines) or next-generation vaccines (e.g., new RNA sequences, mucosal vaccines) will be required to deal with SARS-CoV-2 evolution. A diverse range of vaccine types are already being used around the globe, and vaccine schedules in many locations are being continually adjusted. If this diversity generates relevant immunological heterogeneity within and among populations, then natural selection could favor different viral variants at different times in different locations, and perhaps even result in the coexistence of several variants. If so, vaccination programs may need to be continually adjusted at a national or regional level, as is necessary to control coronaviruses in agriculture [78,79]. The more that vaccination suppresses transmission, targets multiple epitopes, and more effectively inhibits infection and within-host replication and so mutation and recombination, the better it will be at slowing the rate of adaptation (Box 2) and providing sustainable long-term efficacy [56].

## Conclusions

In the early phase of pandemics, we expect the rise of variants that are better at spreading than their ancestors in both naïve and primed hosts (generalists). Later on, pathogen evolution should involve specialized adaptations to primed hosts and so some decrease of adaptation to naïve hosts. Both generalist and specialist variants can be inhibited by immunity, where the growth rate of infections decreases as the fraction of primed hosts increases. Under these circumstances, even if the impact of vaccination is eroded by pathogen evolution, the overall spread of infection is still reduced by vaccination. Immunity-facilitated variants can also arise. In this case, the overall spread of infection could theoretically go up as the fraction of primed hosts increases (such as through vaccination) but this does not imply that the overall level of disease necessarily will increase in either an individual infection or in the population overall.

Although our framework predicts the direction and strength of selection, it does not precisely predict the evolutionary trajectory that will be followed because there is no way of knowing in advance what phenotypes are available to the pathogen genetically (via mutation or recombination). There is also no way of knowing in advance how particular mutations relate to the multiple dimensions of the fitness landscape, even if they may have an advantage on a particular dimension in a laboratory assay.

So far, the SARS-CoV-2 variants of concern that have become dominant have been immunity-inhibited. Many of these variants are also generalists that would have spread regardless of vaccination. At some point, we expect further adaptation to result from the spread of specialist variants, although whether these variants will be immunity-inhibited or immunity-facilitated will depend on mutational availability. Beyond these expectations, a priori prediction about future vaccine efficacy and disease severity for SARS-CoV-2 is not possible. Molecular epidemiological surveillance will be critical for detecting and characterizing viral adaptation as it unfolds.

## Supporting information

S1 Appendix Derivation of the model and framework. (DOCX)

S2 Appendix Alternative frameworks and definitions. (DOCX)

S1 Fig Typology of pathogen variants. We can identify 8 different types of variants. The panel (a) is expanding the description of Fig 2 and the panel (b) is indicating the location of these 8 types. Variant type I is adapted to naïve hosts but maladapted on primed hosts. Variant type V is maladapted on both types of hosts. We focus on the 6 immunity-adapted variants with Δ*r_P_*>0. Variants II, III, and IV are generalist variants (i.e., *r_i,N_*>0) and the magnitude of Δ*r_P_* explains the difference between these 3 variants. Variants VI, VII, and VIII are specialist variants (i.e., Δ*r_N_*<0) and the magnitude explains the difference between these 3 variants. Note that variants IV, VII, and VIII have a growth rate that increases with the fraction of hosts primed. This increased growth rate can have major public health implications. In particular, with variants IV and VIII, evolution is expected to yield a higher pathogen growth rate after 100% primed (the evolved growth rate *r_i,P_* is indicated with the black dot) than after 0% primed (the evolved growth rate *r_i,N_* indicated with the white dot).
(TIF)
