# Supplementary material for: Pathogen evolution during vaccination campaigns
Source: PLoS Biol. 2022 Sep 23;20(9):e3001804. doi: 10.1371/journal.pbio.3001804 (PMC9553060; doi:10.1371/journal.pbio.3001804)
Supplement: S1 Appendix — (DOCX) [file pbio.3001804.s001.docx]

**S1 Appendix**

In this appendix we derive the expressions presented in the main text, and we also show how changes in the three main components of fitness of a variant affect the evolutionary dynamics, and that the classification into variants shown in **Fig 2** remains consistent regardless of epidemiological conditions.

**1. The model**

In general the reproductive success of a variant is determined by three main components of fitness: (i) *infectivity* - the probability that, upon exposure, a variant infects either type of host; (ii) *transmissibility* - the rate at which a variant produces infectious propagules from either host type that contact uninfected individuals; and (iii) *the infectious period*  – the time period during an infection in each host type when a variant produces infectious propagules. All else equal, variants with increased infectivity or increased transmissibility will have an increased growth rate. To understand the role of the infectious period on reproductive success, suppose $\tau$ denotes the amount of time that has elapsed since the start of an infection and $\phi\left( \tau\right)d\tau$ denotes the fraction of all new infections that this single infection generates in the time interval $(t,t+dt)$. Then, all else equal, variants for which $\phi\left( \tau\right)d\tau>0$ for long period of time (i.e., long infection durations) and variants for which the bulk of the density of $\phi\left( \tau\right)$ is concentrated early in the infection (i.e., short generation intervals) will have an increased growth rate. In the model that follows for simplicity we assume that $\phi\left( \tau\right)$ follows an exponential distribution but this assumption is not required for the general considerations of the main text, nor for the categorization of variants presented in Figs 2-4.

We track the dynamics of variant $i$ in a host population with a density $S_{N}$ of naïve hosts and a density $S_{P}$ of primed hosts using the following system of differential equations:

$$\dot{I}_{i}^{N}=h_{i}\sigma_{i}^{N}S_{N}-\gamma_{i}^{N}I_{i}^{N}$$

$$\dot{I}_{i}^{P}=h_{i}\sigma_{i}^{P}S_{V}-\gamma_{i}^{P}I_{i}^{P}$$

$$h_{i}=\beta_{i}^{N}I_{i}^{N}+\beta_{i}^{P}I_{i}^{P}$$

The reproductive success of a variant is determined by the three components of fitness:

- $\sigma_{i}^{N}$ and $\sigma_{i}^{P}$: the infectivity of variant $i$ in naïve and primed hosts
- $\beta_{i}^{N}$ and $\beta_{i}^{P}$: the transmission rate of variant $i$ from naïve and primed hosts
- $\gamma_{i}^{N}$ and $\gamma_{i}^{P}$: the infectious period of variant $i$ in naïve and primed hosts

Below we present the derivation of the three dynamical variables mentioned in **Box 3** that capture the epidemiological and evolutionary dynamics during adaptation (equations (S1), (S2) and (S3) below).

**2. The growth rate of the epidemic**

If the change in the density of susceptible hosts occurs slowly relative to the spread of infection then the per capita growth rate of infections will be given by the dominant eigenvalue $r_{i}$ of the matrix:

$$\mathbf{R}_{i}=\left( \begin{matrix} r_{i}^{NN} & r_{i}^{PN} \\ r_{i}^{NP} & r_{i}^{PP} \end{matrix} \right)$$

where

$$r_{i}^{NN}=\beta_{i}^{N}\sigma_{i}^{N}S_{N}-\gamma_{i}^{N}$$

$$r_{i}^{PN}=\beta_{i}^{P}\sigma_{i}^{N}S_{N}$$

$$r_{i}^{PP}=\beta_{i}^{P}\sigma_{i}^{P}S_{P}-\gamma_{i}^{P}$$

$$r_{i}^{NP}=\beta_{i}^{N}\sigma_{i}^{P}S_{P}$$

If we further define $\delta_{i}=\gamma_{i}^{P}-\gamma_{i}^{N}$ then we can write

$$r_{i}=\left( 1-p \right)r_{i,N}+pr_{i,P}-\frac{S_{P}\beta_{i}^{P}\sigma_{i}^{P}}{S_{N}\beta_{i}^{N}\sigma_{i}^{N}+S_{P}\beta_{i}^{P}\sigma_{i}^{P}}\delta_{i}+{O[\delta_{i}]}^{2}$$

with: $r_{i,N}=S\beta_{i}^{N}\sigma_{i}^{N}-\gamma_{i}^{N}$, $r_{i,P}=S\beta_{i}^{P}\sigma_{i}^{P}-\gamma_{i}^{P}$, and where $S{=S}_{N}+S_{P}$ and $p=\frac{S_{P}}{S_{N}+S_{P}}$ is the *coverage of vaccination* (i.e., the fraction of the uninfected population that is vaccinated).

When $\delta_{i}=0$ this simplifies to:

$$r_{i}=\left( 1-p \right)r_{i,N}+pr_{i,P}$$

which is equation (1) of the main text. This then leads to the typology in **Fig 2** (and the more exhaustive typology in **S1 Fig**). Notice, however, that the categorization of variants in **Fig 2** is not specific to this model but instead can be applied much more generally. In principle we can always quantify the per capita growth rate of a variant in a fully naïve and a fully primed population no matter what the epidemiological model or parameter values. The simplification that $\delta_{i}=0$ is made entirely for expositional purposes since it allows one to write the overall per capita growth rate of a variant for any fraction of primed hosts *p* as a convex combination of the variant’s growth rate in each of the two “pure” populations. As such the growth rates in **Fig 2** are connected simply by straight lines. Furthermore, the kind of categorization in **Fig 2** can be applied regardless of the epidemiological state of the population (e.g., exponential growth, endemicity, etc.; see Section 5 below). For example, as the epidemic grows the density of uninfected hosts will drop and perhaps non-pharmaceutical interventions will be implemented to reduce the force of infection. Although both processes will reduce the absolute growth rates of all variants we can nevertheless construct a version of **Fig 2** for any epidemiological context of interest. Moreover, because the categorization of variants in **Fig 2** depends only on their *relative* growth rates, if this relative ordering does not change with epidemiological context then a variant’s classification into one of the four categories will remain consistent regardless of what is happening epidemiologically.

In the following, for simplicity, the effects of the mutation on the different viral components of fitness in host $X$ (where $X=N$ or $P$) will be assumed to be small and will be denoted:

- $\Delta\beta_{X}=\beta_{m}^{X}-\beta_{w}^{X}$
- $\Delta\sigma_{X}=\sigma_{m}^{X}-\sigma_{w}^{X}$
- $\Delta\gamma_{X}=\gamma_{m}^{X}-\gamma_{w}^{X}$

and the components of fitness of the wildtype will be noted:

- $\delta=\delta_{w}$
- $\beta_{X}=\beta_{w}^{X}$
- $\sigma_{X}=\sigma_{w}^{X}$
- $\gamma_{X}=\gamma_{w}^{X}$

Using this notation the growth rate of the wildtype population is:

$$r=\left( 1-p \right)r_{N}+pr_{P}$$

and the growth rate of the novel variant is $r+\Delta r$ where:

$$\Delta r=\left( 1-p \right)\underset{\Delta r_{N}}{\underbrace{\left( S\left( {\Delta\beta}_{N}\sigma_{N}+\beta_{N}\Delta\sigma_{N} \right)-\Delta\gamma_{N} \right)}}+p \underset{\Delta r_{P}}{\underbrace{\left( S\left( {\Delta\beta}_{P}\sigma_{P}+\beta_{P}\Delta\sigma_{P} \right)-\Delta\gamma_{P} \right)}}$$

The growth rate of the whole population of all infected individuals is simply:

| $\bar{r}=r+f_{m}\Delta r$ | (S1) |
| --- | --- |

where $f_{m}$ is the frequency of the novel variant:

$$f_{m}=\frac{I_{m}^{N}+I_{m}^{P}}{I_{w}^{N}+I_{w}^{P}+I_{m}^{N}+I_{m}^{P}}$$

Thus, a variant with a higher growth rate will spread when $\Delta r>0$ and the subsequent increase in mutant frequency will affect the growth rate of the whole pathogen population.

**3. The dynamics of variant frequency**

The dynamics of the variant frequency $f_{m}$ depends on the distribution of the variant in naïve and primed hosts [1]. But if the phenotype of the variant is not very different from that of the wildtype we can obtain a very good approximation of these dynamics using [2]:

$$\dot{f}_{m}\approx f_{m}\left( 1-f_{m} \right)\mathbf{V}^{T}\Delta\mathbf{R}_{m}\mathbf{F}$$

where $\mathbf{V}^{T}$is the vector of reproductive values and $\mathbf{F}$ is the vector of class frequencies which correspond to the conormalised (i.e. $\mathbf{V}^{T}\mathbf{F=1}$) left and right eigenvectors of $\mathbf{R}_{w}$, respectively:

$$\boldsymbol{F\propto}\left\{ \frac{S_{N}\sigma_{N}}{S_{P}\sigma_{P}}\left( 1+\frac{\delta}{S_{N}\beta_{N}\sigma_{N}+S_{P}\beta_{P}\sigma_{P}} \right)\mathbf{+}O\left( \delta^{2} \right),1 \right\}$$

$$\mathbf{V}^{T}\propto\left\{ \frac{\beta_{N}}{\beta_{P}}\left( 1+\frac{\delta}{S_{N}\beta_{N}\sigma_{N}+S_{P}\beta_{P}\sigma_{P}} \right)\mathbf{+}O\left( \delta^{2} \right),1 \right\}$$

and selection on the different transitions is given by:

$$\Delta\mathbf{R}_{m}=\left( \begin{matrix} s_{NN} & s_{PN} \\ s_{NP} & s_{PP} \end{matrix} \right)$$

where:

$s_{NN}=\left( \Delta\beta_{N} \sigma_{N}+\beta_{N} \Delta\sigma_{N} \right) S_{N}-\Delta\gamma_{N}$ Selection coefficient (when $N$ infect $N$)

$s_{NP}=\left( \Delta\beta_{N} \sigma_{P}+\beta_{N} \Delta\sigma_{P} \right) S_{P}$ Selection coefficient (when $N$ infect $P$)

$s_{PP}=\left( \Delta\beta_{P} \sigma_{P}+\beta_{P} \Delta\sigma_{P} \right) S_{P}-\Delta\gamma_{P}$ Selection coefficient (when $P$ infect $V$)

$s_{PN}=\left( \Delta\beta_{P} \sigma_{N}+\beta_{P} \Delta\sigma_{N} \right) S_{N}$ Selection coefficient (when $P$ infect $N$)

After some calculation this yields:

$$\dot{f}_{m}\approx f_{m}\left( 1-f_{m} \right)s$$

where:

$$s\propto\left( 1-p \right)S\left( \beta_{N}{\Delta\sigma}_{N}+\sigma_{N}{\Delta\beta}_{N} \right)+pS\left( \beta_{P}{\Delta\sigma}_{P}+\sigma_{P}{\Delta\beta}_{P} \right)-\left( 1-q \right){\Delta\gamma}_{N}-q{\Delta\gamma}_{P}+ \delta K\mathbf{+}O\left( \delta^{2} \right)$$

$$q=\frac{S_{P}\beta_{P}\sigma_{P}}{S_{N}\beta_{N}\sigma_{N}+S_{P}\beta_{P}\sigma_{P}}$$

$$K=\frac{S_{N}S_{P}\beta_{N}\beta_{P}\sigma_{N}\sigma_{P}}{\left( S_{N}\beta_{N}\sigma_{N}+S_{P}\beta_{P}\sigma_{P} \right)^{2}}\left( \left( \frac{{\Delta\beta}_{N}}{\beta_{N}}-\frac{{\Delta\beta}_{P}}{\beta_{P}} \right)+\left( \frac{\Delta\sigma_{N}}{\sigma_{N}}-\frac{\Delta\sigma_{P}}{\sigma_{P}} \right)-\frac{2}{\left( S_{N}\beta_{N}\sigma_{N}+S_{P}\beta_{P}\sigma_{P} \right)}({\Delta\gamma}_{N}-{\Delta\gamma}_{P}) \right)$$

When $\delta=0$ this simplifies as:

| $s\propto\underset{selection in naive hosts}{\underbrace{\left( 1-p \right)S\left( \beta_{N}{\Delta\sigma}_{N}+\sigma_{N}{\Delta\beta}_{N} \right)-\left( 1-q \right){\Delta\gamma}_{N}}}+\underset{selection in primed hosts}{\underbrace{pS\left( \beta_{P}{\Delta\sigma}_{P}+\sigma_{P}{\Delta\beta}_{P} \right)-q{\Delta\gamma}_{P}}}$ | (S2) |
| --- | --- |

Note how selection for higher values of transmission $\beta$ and infectivity $\sigma$ depend on the density of susceptible hosts $S$while selection on the infectious period $\gamma$ does not [3].

**4. The dynamics of differentiation**

Next, we use [1] to track the difference in variant frequency between primed and naïve hosts. The dynamics of variant frequencies in naïve and primed hosts in a well-mixed population is:

$$\dot{f}_{m}^{N}=v_{N}s_{NN}+v_{P}\frac{I_{P}}{I_{N}}s_{PN}+\frac{I_{P}}{I_{N}}\bar{r}_{PN}D$$

$$\dot{f}_{m}^{P}=v_{P}s_{PP}+v_{N}\frac{I_{N}}{I_{P}}s_{NP}-\frac{I_{N}}{I_{P}}\bar{r}_{NP}D$$

where:

$D=f_{m}^{P}-f_{m}^{N}$ Differentiation

$v_{N}=f_{m}^{N}\left( 1-f_{m}^{N} \right)$ Genetic variance in naive hosts

$v_{P}=f_{m}^{P}\left( 1-f_{m}^{P} \right)$ Genetic variance in primed hosts

$$\bar{r}_{PN}=f_{m}^{P}\left( \left( \beta_{P}+\Delta\beta_{P} \right)\left( \sigma_{N}+\Delta\sigma_{N} \right) S_{N} \right)+\left( 1-f_{m}^{P} \right)\left( \beta_{P}\sigma_{N} S_{N} \right)=\beta_{P}\sigma_{N} S_{N}+f_{m}^{P}\left( \beta_{P}\Delta\sigma_{N} +\Delta\beta_{P}\sigma_{N} \right)S_{N}=\beta_{P}\sigma_{N} S_{N}+f_{m}^{P}s_{PN}$$

$$\bar{r}_{NP}=f_{m}^{N}\left( \left( \beta_{N}+\Delta\beta_{N} \right) \left( \sigma_{P}+\Delta\sigma_{P} \right) S_{P} \right)+\left( 1-f_{m}^{N} \right)\left( \beta_{N} \sigma_{P} S_{P} \right)=\beta_{N} \sigma_{P} S_{P}+f_{m}^{N}\left( \beta_{N}\Delta\sigma_{P} +\Delta\beta_{N}\sigma_{P} \right)S_{P}=\beta_{N} \sigma_{P} S_{P}+f_{m}^{N}s_{NP}$$

The dynamics of differentiation $D$ is therefore given by:

$$\dot{D}=v_{P}\left( s_{PP}-\frac{I_{P}}{I_{N}}s_{PN} \right)-v_{N}\left( s_{NN}-\frac{I_{N}}{I_{P}}s_{NP} \right)-D\left( \frac{I_{N}}{I_{P}}\bar{r}_{NP}+\frac{I_{P}}{I_{N}}\bar{r}_{PN} \right)$$

If we assume there is no differentiation initially ($D=f_{m}^{P}-f_{m}^{N}=0$, which also means genetic variance is the same in the two environments, $v_{N}=v_{P}=v=f_{m}\left( 1-f_{m} \right)$ then the dynamics of differentiation are:

$$\dot{D}=v\left( s_{PP}-s_{NN}-\frac{I_{P}}{I_{N}}s_{PN}+\frac{I_{N}}{I_{P}}s_{NP} \right)$$

$$\dot{D}=v\left( \left( \Delta\beta_{P} \sigma_{P}+\beta_{P} \Delta\sigma_{P} \right) S_{P}-\Delta\gamma_{P}-\left( \Delta\beta_{N} \sigma_{N}+\beta_{N} \Delta\sigma_{N} \right) S_{N}+\Delta\gamma_{N}-\frac{I_{P}}{I_{N}}\left( \Delta\beta_{P} \sigma_{N}+\beta_{P} \Delta\sigma_{N} \right) S_{N}+\frac{I_{N}}{I_{P}}\left( \Delta\beta_{N} \sigma_{P}+\beta_{N} \Delta\sigma_{P} \right) S_{P} \right)$$

If we further assume that the prevalence is low so that $S_{N}$ and $S_{P}$ remains constant during the early stage of the epidemic the prevalence will grow exponentially and the ratio $\frac{I_{N}}{I_{P}}$ will remain constant. The value of this ratio can be computed from the vector $\mathbf{F}$ of class frequencies given above:

$$\frac{I_{N}}{I_{P}}=\frac{S_{N}\sigma_{N}}{S_{P}\sigma_{P}}\left( 1+\frac{\delta}{S_{N}\beta_{N}\sigma_{N}+S_{P}\beta_{P}\sigma_{P}} \right)+O\left( \delta^{2} \right)$$

The dynamics of differentiation therefore becomes:

$$\dot{D}=v\left( \left( S_{N}\beta_{N}\sigma_{N}+S_{P}\beta_{P}\sigma_{P} \right)\left( \frac{\Delta\sigma_{P}}{\sigma_{P}}- \frac{\Delta\sigma_{N}}{\sigma_{N}} \right)+\Delta\gamma_{N}-\Delta\gamma_{P}+\frac{s_{NP}\frac{\sigma_{P}S_{P}}{\sigma_{N}S_{N}}+s_{PN}\frac{\sigma_{N}S_{N}}{\sigma_{P}S_{P}}}{S_{N}\beta_{N}\sigma_{N}+S_{P}\beta_{P}\sigma_{P}}\delta\right)+O\left( \delta^{2} \right)$$

When $\delta=0$ this simplifies as:

| $\dot{D}=v\left( S\left( \left( 1-p \right)\beta_{N}\sigma_{N}+p\beta_{P}\sigma_{P} \right)\left( \frac{\Delta\sigma_{P}}{\sigma_{P}}- \frac{\Delta\sigma_{N}}{\sigma_{N}} \right)-\left( \Delta\gamma_{P}-\Delta\gamma_{N} \right) \right)$ | (S3) |
| --- | --- |

Note how differentiation is not driven by the transmission rates of the mutant but by its relative infectivity in naïve and primed hosts.

**5. Generalizing the Model**

To see that the above results can be applied to a broader set of epidemiological circumstances, consider a model where there is a single variant and *n* different immunological host types (we can think of the latter as hosts that differ in their vaccination history and/or their infection history, and so differ in the extent to which the variant might infect them). This is a generalization of the model in the manuscript and it can be written as

$$\dot{S}=G\left( S,I \right)$$

$$\dot{I}=\left( F(S)-V(S) \right)I$$

Here *S* is a vector of densities of uninfected hosts of each type and *I* is a vector of densities of infected hosts of each type. The function *G* specifies the rate of change of each of the uninfected host types. The function *F*(*S*) is an *n* x *n* matrix specifying how each infected host type generates infections of all the *n* different host types, and *V*(*S*) is an *n* x *n* matrix giving the loss rates of infection of each of the different host types. In most common situations *V*(*S*) will be a diagonal matrix, independent of *S*.

The main assumption of our framework and categorization is that, for any given epidemiological state (as specified by (*S,I*)), we can measure the per capita growth rate of a variant. Mathematically, this does not require that the naïve population be constant nor that the population of infections is growing exponentially. All that is required is that we can assign a unique per capita growth rate to the variant for each epidemiological state. To see how this can be done, first note that the rate of change of infections of host type *i* is given as $\dot{I}_{i}=\sum_{j} F_{ij}\left( S \right)I_{j}-V_{ii}\left( S \right)I_{i}$. Now define $I_{T}=\sum_{i} I_{i}$ as the total density of infected hosts of all types. Then the rate of change of $I_{T}$ is given by $\dot{I}_{T}=r\left( S,I \right)I_{T}$ where $r\left( S,I \right)=\frac{\sum_{ij} F_{ij}\left( S \right)I_{j}-\sum_{i} V_{ii}\left( S \right)I_{i}}{\sum_{i} I_{i}}$ is the per capita growth rate of the variant, which depends on epidemiological state (*S,I*). We can also see how this general result relates to the special case where the population of infections is growing exponentially. In this case the fraction of infections in each host type will be independent of time and so we can simplify things further since $r\left( S,I \right)$ will then be independent of *I*.

How does this relate to Fig 1, on which the entire categorization is based? First, Fig 1 assumes there are only 2 host types – naïve and primed. Therefore, we can characterize the vector S by two components; $S_{N}$ and $S_{P}$. In Fig 1 we instead use the parameterization $S_{N}=S_{Total} (1-p)$ and $S_{P}=S_{Total} p$), where $S_{Total}$ is the total number of uninfected hosts of both types and *p* is the fraction of this total that are primed. Then, on the left side of each panel in Fig 1, we have *p =* 0*,* and on the right side we have *p =* 1. The remainder of the plot is specific to the epidemiological state ($S_{Total},I)$. To put this another way, the dots in Fig 1 are the growth rates that the variants would have in a population that is either 100% naïve or 100% vaccinated, but specific to a particular epidemiological state ($S_{Total},I)$. Thus, as epidemiological feedbacks occur, $S_{Total}$and *I* will change, and so the points in Fig 1 will move up and/or down. Nevertheless, the categorization which is based on this figure can be applied for any epidemiological state ($S_{Total}$,*I*), and no assumption of any kind about the nature of the epidemiological dynamics is required (we note however, that in Fig 1 we have used straight lines to connect points for each variant and this does require some additional assumptions). Furthermore, if the relative position of the points in Fig 1 remain unchanged as ${(S}_{Total},I)$ changes, then the category in which a variant falls will remain the same regardless of the epidemiological state of the population.

**References:**

[1] Gandon S. & Day T. (2007). The evolutionary epidemiology of vaccination. Journal of the Royal Society Interface. 4: 803-817.

[2] Otto, S. P., & Day, T. (2011). A biologist's guide to mathematical modeling in ecology and evolution. Princeton University Press.

[3] Day T., Gandon S., Lion S. & Otto S. (2020). [On the evolutionary epidemiology of SARS-CoV-2.](http://dx.doi.org/10.1016/j.cub.2020.06.031) Current Biology. 30(15): R849-R857.
