# Supplementary material for: Pathogen evolution during vaccination campaigns
Source: PLoS Biol. 2022 Sep 23;20(9):e3001804. doi: 10.1371/journal.pbio.3001804 (PMC9553060; doi:10.1371/journal.pbio.3001804)
Supplement: S2 Appendix — (DOCX) [file pbio.3001804.s002.docx]

**S2 Appendix**

In **Figs 2-4** we use the per capita growth rate, *r*, of infections in a fully naïve and a fully primed population as the axes for visualizing different variants. Another common measure of the fitness of a variant is its reproduction number, $R_{0}$ and the effective reproduction number $R$. The reproduction number $R_{0}$­ is a dimensionless quantity that gives the number of new infections produced by a single infected individual in a fully susceptible population. The effective reproduction number is analogous but it also takes into account any depletion of the susceptible population as well as the effects of any control measures. Neither $R_{0}$ nor $R$ account for the timing of when these infections occur, however, and so they do not provide information about the timeframe over which infections are spreading through a population. The per capita growth rate has units 1/time and so it does account for the timeframe of infection spread. Two variants can therefore have different values of $r$ even if they each produce the same total number of new infections per infection (i.e., $R_{0}$ or $R$), if they differ in how the production of these new infections is spread out over time.

There are several alternative ways one might construct plots analogous to that of **Figs 2-4**. One is simply to use a variant’s reproduction number in each of the two host types as the measure of fitness rather than its per capita growth rate. However, doing so has two disadvantages. First, differences between variants in the timing of transmission will affect their relative competitive abilities during a vaccination campaign and this is not accounted for when using the reproduction number as a measure of fitness (for the reasons discussed above). Second, as has become clear with the appearance and spread of novel variants during the SARS-CoV-2 pandemic, one of the first and easiest quantities estimated for a variant is its selection coefficient (i.e., the difference in growth rate between it and the current dominant type: $s=r_{new}-r_{old}$ ). Such estimates are all that is required to place a new variant on a plot like **Figs 2-4**.

Another alternative to the axes used in **Fig 3** would be to use a variant’s overall fitness across both host types as one axis (as either $r$ or $R_{0}$) and its ability to evade vaccine-induced immunity as the other. The latter is often quantified by the vaccine efficacy against the variant, as measured by ${VE}_{i}=1-\frac{\sigma_{i}^{P}}{\sigma_{i}^{N}}$, where $\sigma_{i}^{N}$ and $\sigma_{i}^{P}$ is the infectivity of the variant in naïve and primed hosts respectively. There are drawbacks with this approach as well. First, the axes in such a plot are not independent. Second, any measure of the overall fitness of a variant will necessarily depend on the fraction of the population that is primed and so a different plot would be needed for different situations. It is possible to circumvent this problem by using the fitness of a variant in the naïve population as one of the axes rather than its overall fitness, but then it is no longer possible to visualize overall fitness on such a plot. The reason is that a combination of a variant’s fitness in naïve hosts and its ${VE}_{i}$ is not sufficient to determine its overall fitness. Differences between variants in how the immunity affects disease severity and transmissibility can also influence a variant’s competitive ability and these are excluded from such a plot (since ${VE}_{i}$ quantifies only the effect on infectivity which is only one of three main components of pathogen fitness, see **Box 1**).
