# Supplementary figures and images for: Pathogen evolution during vaccination campaigns
Source: PLoS Biol. 2022 Sep 23;20(9):e3001804. doi: 10.1371/journal.pbio.3001804 (PMC9553060; doi:10.1371/journal.pbio.3001804)

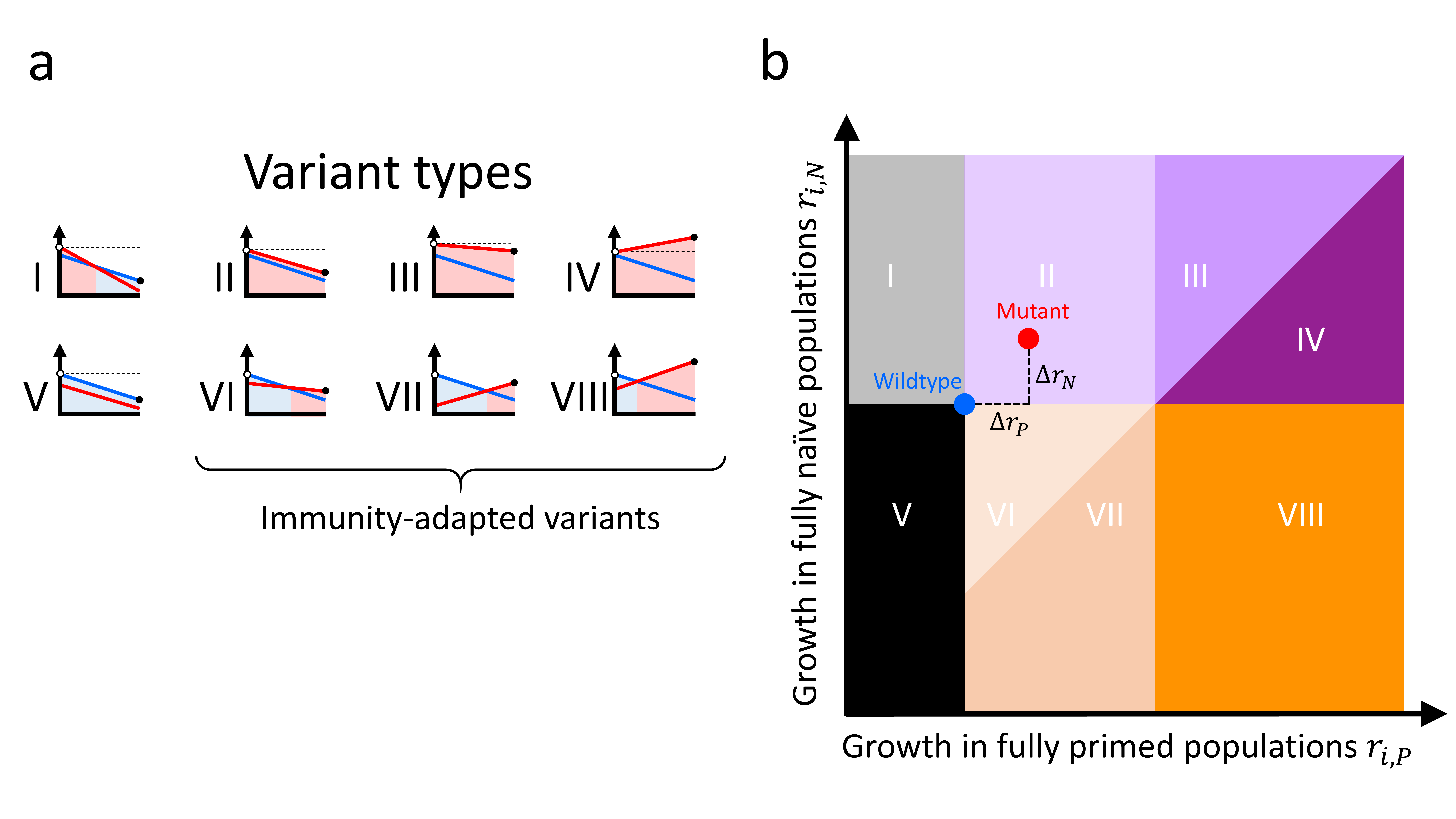

Supplement: S1 Fig — We can identify 8 different types of variants. The panel (a) is expanding the description of Fig 2 and the panel (b) is indicating the location of these 8 types. Variant type I is adapted to naïve hosts but maladapted on primed hosts. Variant type V is maladapted on both types of hosts. We focus on the 6 immunity-adapted variants with ΔrP>0. Variants II, III, and IV are generalist variants (i.e., ri,N>0) and the magnitude of ΔrP explains the difference between these 3 variants. Variants VI, VII, and VIII are specialist variants (i.e., ΔrN<0) and the magnitude explains the difference between these 3 variants. Note that variants IV, VII, and VIII have a growth rate that increases with the fraction of hosts primed. This increased growth rate can have major public health implications. In particular, with variants IV and VIII, evolution is expected to yield a higher pathogen growth rate after 100% primed (the evolved growth rate ri,P is indicated with the black dot) than after 0% primed (the evolved growth rate ri,N indicated with the white dot). (TIF) [file pbio.3001804.s003.tif]
